# Prognostic Factors of Dysphagia and Recovery Following Pediatric Acquired Brain Injury

**DOI:** 10.3390/children13020301

**Published:** 2026-02-21

**Authors:** Suhad Bishara, Oshrat Sella Weiss, Saja Hejla-Assi, Tmira Nachum, Sharon Shaklai

**Affiliations:** 1 Speech and Language Department, Loewenstein Rehabilitation Medical Center, Raanana 4355840, Israel; 2 Department of Communication Disorders, Steyer School of Health Professions, Gray Faculty of Medical and Health Sciences, Tel-Aviv University, Tel-Aviv 6997801, Israel; 3 Department of Communication Sciences and Disorders, University of Haifa, Haifa 3103301, Israel; oshratse@clalit.org.il; 4 Department of Audiology and Speech and Language, Schneider Children’s Medical Center, Petah Tikva 4920235, Israel; 5 Audiology Speech and Language Pathology Department, Rabin Medical Center, Petah Tikvah 4941492, Israel; 6 Gray Faculty of Medical and Health Sciences, Tel-Aviv University, Tel-Aviv 6997801, Israel; 7 Pediatric and Youth Rehabilitation Department, Loewenstein Rehabilitation Medical Center, Raanana 4355840, Israel

**Keywords:** dysphagia, acquired brain injury, pediatric rehabilitation, swallowing recovery, consciousness, cranial nerve

## Abstract

**Highlights:**

**What are the main findings?**

- 61% of children with acquired brain injury (ABI) presented with dysphagia.
- Recovery rate was 78% with assigned treatment.
- Reduced consciousness state, cranial nerve involvement, voice disorders, and etiology of CNS tumors were associated with dysphagia post ABI.
- Recovery of dysphagia was associated with conscious state at admission and severity of dysphagia

**What are the implications of the main findings?**

- All children with ABI should be evaluated for dysphagia at admission to rehabilitation.
- Recovery rate of dysphagia in children with acquired brain injury with assigned treatment is high.

**Abstract:**

Objectives: Dysphagia is a major complication of acquired brain injury (ABI) in children; however, its trajectory and prognostic indicators remain insufficiently characterized. This study aimed to identify predictors of dysphagia and its recovery following pediatric ABI. Methods: This retrospective study included all children admitted with ABI to tertiary pediatric rehabilitation center between 2014 and 2017. Data were collected from electronic medical records. Results: One hundred children aged 2:00–17:11 years were included; 61% had dysphagia at admission. Participants with dysphagia received speech–language pathology (SLP) treatment, with a recovery rate of 78.68%. Treatment duration was significantly shorter among children who recovered (36 days) compared with those who did not (136 days; *p* < 0.001). Dysphagia at admission was associated with mechanical ventilation, duration of unconsciousness, duration of acute hospitalization, CNS tumor etiology, cranial nerve impairment (V, IX, X, XII), voice and speech impairments, and cognitive and behavioral impairments. Logistic regression showed that reduced consciousness, cranial nerve impairment, voice disorders, and CNS tumors explained 70.6% of dysphagia likelihood. Non-recovery was associated with unconsciousness, enteral feeding, hypoglossal injury, and dysphagia severity at admission. Level of consciousness at admission explained 33.7% of recovery likelihood. Conclusions: Dysphagia was highly prevalent among children with ABI. Recovery rates following SLP treatment were high and were associated with level of consciousness at admission to rehabilitation.

## 1. Introduction

Acquired brain injury (ABI) refers to brain damage occurring after birth and unrelated to hereditary or progressive conditions. ABI can result from traumatic causes such as traumatic brain injury (TBI), or non-traumatic etiologies, including stroke, tumors, infections, autoimmune disorders, or asphyxia [1]. In adults, ABI most commonly results from cerebrovascular accidents, while in adolescents and young adults, TBI-induced ABI is more prevalent [2]. Importantly, the clinical presentation and outcomes of ABI in children are highly heterogeneous and depend largely on injury etiology and severity rather than age alone. In pediatric populations, recovery trajectories in relation to age and etiology are not yet clearly defined. Younger age does not uniformly predict better functional outcomes, particularly following severe traumatic or hypoxic brain injury [3].

In adults, dysphagia is a common consequence of ABI and may affect the oral and/or pharyngeal phases of swallowing [1]. Prevalence ranges from 27% to 80%, reaching up to 93% in cases of severe TBI [4,5,6,7]. Kumar et al. reported stroke as a risk factor for dysphagia, with an odds ratio (OR) of 21.83 [4]. Dysphagia in adults leads to serious complications including aspiration, pneumonia, dehydration, malnutrition, and even death. Dysphagia has been found to be associated with increased morbidity, prolonged hospital stays, and elevated healthcare costs [5,6,7].

Several prognostic factors for persistent dysphagia and reduced recovery have been identified in adult populations. These include older age [8], greater neurological severity [9], cognitive impairment [10], initial dysphagia severity [11], delayed initiation of oral feeding [12,13], prolonged mechanical ventilation [12], aspiration, and longer acute hospitalization [14]. In stroke patients, the location of brain stem or bi-hemispheric injuries [15] and severity [4] as indicated by higher National Institutes of Health Stroke Scale scores (≥12), correlated with persistence of dysphagia. Conversely, certain protective factors have been shown to support a more favorable outcome. Time to first swallowing assessment [12] and early swallowing intervention [15] have been associated with improved prognosis. Additionally, preserved post stroke functional status has been linked to better recovery [15].

In contrast to the extensive literature on dysphagia in adult populations, data in pediatric populations remain limited. Morgan et al. [16] reported 3.8% incidence of dysphagia following pediatric TBI, which increased with severity of injury. Following severe TBI, the prevalence of dysphagia in pediatric populations was reported at 68%, while it was only 15% and 1% following moderate and mild TBI, respectively [16,17,18]. Similar to adults, in children with TBI, dysphagia was associated with longer hospital stays, increased need for ventilation, supplemental feeding, and greater caregiver burden.

In acute pediatric stroke, Sherman et al. [19] reported that up to 41% of patients had dysphagia. They reported that post stroke dysphagia increased with higher scores on the Pediatric National Institutes of Health Stroke Scale and with the involvement of middle cerebral artery territory. Of children diagnosed with dysphagia in the acute phase, 42% were discharged with persistent dysphagia. However, these studies referring to post-pediatric TBI [16,17] and stroke [19] populations primarily focused on the acute hospitalization phase.

Addressing the gap in data on pediatric dysphagia in the subacute phase is essential for improving early risk stratification and guiding clinical decision-making for rehabilitative care. Considering the above, the aims of the present study were (1) to identify factors associated with the presence of dysphagia at admission to a pediatric rehabilitation center following ABI; and (2) to examine predictors of dysphagia recovery following speech–language pathology intervention. We hypothesized that clinical and etiological factors would be associated with both the presence of and the recovery from dysphagia, potentially informing prognosis and individualized rehabilitation strategies.

## 2. Materials and Methods

### 2.1. Study Design

This was a retrospective historical cohort study conducted at the Pediatric Rehabilitation Department of the Loewenstein Rehabilitation Medical Center. All participants admitted to this department between 2014 and 2017 were screened for eligibility.

### 2.2. Inclusion Criteria

Participants were included in the study if they met both of the following inclusion criteria: 1. Diagnosed with ABI, and 2. Aged between 2:00 and 17:11 months at the time of admission to rehabilitation. The reason for excluding children under the age of two years was that the swallowing mechanism still undergoes developmental maturation during the first two years of life [20].

### 2.3. Exclusion Criteria

Participants were excluded from participation in the study if they met at least one of the following exclusion criteria: (1) premorbid dysphagia prior to ABI; (2) documented developmental delay before ABI; (3) death during rehabilitation due to causes unrelated to dysphagia; or 4. incomplete or missing medical records.

### 2.4. Subject Population

**Figure 1:**
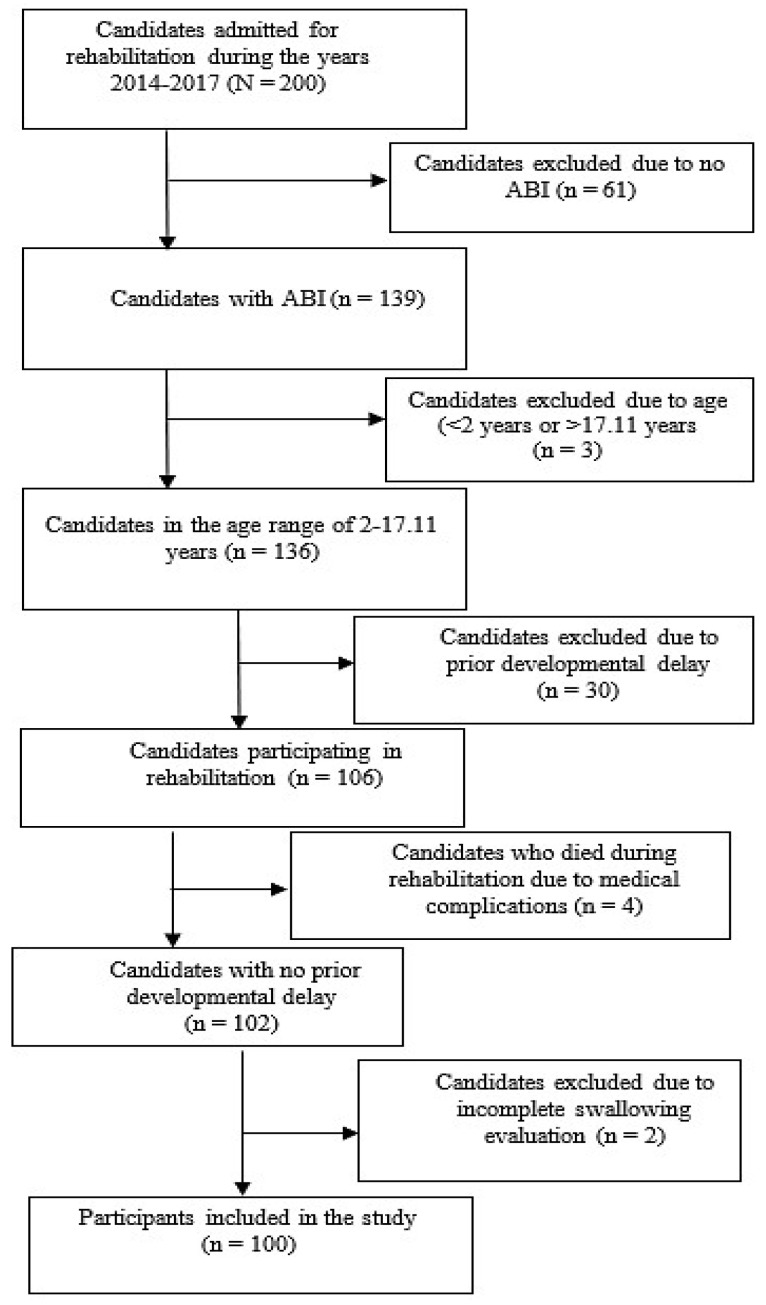
Schematic presentation of candidates screened for the study, candidates excluded from the study (including reasons for exclusion), and participants included in the study. ABI = acquired brain injury.

A total of 200 candidates were admitted for rehabilitation from 2014 to 2017. Of these, 100 candidates met all inclusion criteria, had a swallowing evaluation and were included in the final study analysis. Figure 1 presents the number of candidates screened; the number of candidates excluded, including reasons for exclusion; and the number of participants who passed the screening procedure and were included in the study.

### 2.5. Study Procedure

All data analyzed in this study was collected from the patients’ electronic medical records. The following data were collected.

Demographic data: age, gender, ethnicity (Jewish/Arab), and etiology of ABI, including TBI, central nervous system (CNS) tumor, stroke, anoxic brain injury (AnBI), infections, and acute disseminated encephalomyelitis (ADEM). For participants with TBI, the Glasgow Coma Scale (GCS) [21] score was recorded upon arrival to acute hospitalization.

Imaging characteristics: location of CNS hemispheric damage (right/left/bilateral).

Acute hospitalization variables: length of acute hospitalization in days; presence of mechanical ventilation and pneumonia, including ventilator-associated pneumonia (yes/no).

Rehabilitation characteristics: length of rehabilitation in days at the tertiary rehabilitation center; neurological impairments upon arrival to rehabilitation, including conscious state (yes/no), cranial nerve involvement (yes/no), hemiparesis/plegia (yes/no), cognitive or behavioral disorders (yes/no), and voice or speech disorders (yes/no).

Participants were divided into two groups according to the presence or absence of dysphagia at admission. Those diagnosed with dysphagia at admission were treated by a speech–language pathologist, either until the dysphagia resolved or, if not resolved, until discharge from the rehabilitation hospitalization. All clinical records from the time of discharge were re-evaluated.

### 2.6. Clinical Dysphagia Assessment

Each participant underwent a comprehensive evaluation by an experienced speech–language pathologist. The assessment included the following items:

- Oromotor structure and function: assessment of lips, tongue, and palate.
- Cranial nerve examination: functional status of cranial nerves trigeminal (V), facial (VII), glossopharyngeal (IX), vagal (X), and hypoglossal (XII), as documented by the admitting physician.
- Ability to swallow saliva.
- Cognitive and communication status: arousal, awareness, impulsivity, and motivation as judged by the attending physician.
- Swallowing trials: evaluation across different textures (puree, soft, regular solids, and varying liquid consistencies), selected based on the child’s clinical abilities.

A diagnosis of dysphagia was given if an impairment was identified in any of the items of the clinical swallowing assessment. Children with significantly reduced saliva control or low arousal states that rendered oral intake unsafe were also classified as having dysphagia.

The severity of dysphagia was stratified to four groups according to the Functional Oral Intake Scale (FOIS) [22], as follows: FOIS 7 = participants without dysphagia, FOIS 6 = participants with mild dysphagia, FOIS 4–5 = participants with moderate dysphagia, FOIS 1–3 = participants with severe dysphagia (tube-dependent).

Speech and language pathologist (SLP) intervention during hospitalization: All participants with dysphagia, including those with reduced conscious state or limited cooperation, received treatment by an SLP until discharge from rehabilitation or dysphagia recovery. The SLP therapy consisted of rehabilitation intervention focusing on improving patients’ sensorimotor functions, including sensory stimulations such as tactile thermal stimulation, oropharyngeal strengthening exercises such as tongue strengthening, effortful swallow and bolus trails. Each participant had a specific intervention program tailored to their function level and ability to actively cooperate in treatment. Treatment goals dynamically changed as the participant advanced during treatment.

Recovery was defined as the absence of dysphagia across all tested textures and phases of swallowing at the time of discharge from rehabilitation.

### 2.7. Statistical Analysis

Characteristics of the two groups—participants with and without dysphagia—are presented as median (interquartile range [IQR]) for non-normally distributed continuous variables, and as frequencies for categorical and ordinal variables. The relationship between categorical variables (e.g., gender, etiology, motor impairment) and the presence of the dependent variables dysphagia, dysphagia severity and recovery from dysphagia were assessed using Chi square tests, and the strength of the relationship was presented by OR with 95% confidence intervals (CIs). For ordinal variables, the relationship with the dependent variables was assessed using Chi square tests and the strength of the relationship was presented by Cramer’s V. To evaluate the normality of continuous variables, both graphical and statistical methods were employed. Specifically, histograms and Normal Q-Q plots were visually inspected, supplemented by the Kolmogorov–Smirnov test to assess the goodness of fit to a normality distribution. The differences in non-normally distributed continuous variables (age, duration of unconsciousness) between patients with and without dysphagia were examined using non-parametric Mann–Whitney U tests and the effect size was assessed using Rank-Biserial Correlation Cohen’s r.

Furthermore, to identify variables that could predict the presence of dysphagia and recovery from dysphagia, multivariable analysis was performed. A Firth logistic regression model was constructed over standard maximum likelihood estimation to reduce bias and handle potential issues of data separation, ensuring the models predicting dysphagia and recovery from dysphagia are more stable given the sample sizes (n = 100 for dysphagia, n = 61 for recovery from dysphagia). Firth’s penalized likelihood approach was specifically chosen since reduced conscious state at admission was spotted only among patients with dysphagia and hence, may almost perfectly predict the outcome, which often leads to biased estimates in standard logistic regression.

The results in the final models are presented as OR with corresponding 95% CIs. The overall model’s fit was evaluated by Chi square’s Likelihood Ratio significance; the percentage of explained likelihood was calculated with Nagelkerke’s R^2^ using the same variables for a logistic regression alongside the model’s discriminative ability by area under curve (AUC), including its 95% CI.

All statistical analyses were performed by a statistician, using an IBM SPSS Statistics software, version 31 (IBM SPSS Inc., Chicago, IL, USA). Statistical significance was defined at *p*-value ≤ 0.05.

### 2.8. Ethics

The study was approved by the Institutional Review Board of the Loewenstein Rehabilitation Medical Center (Approval No. LOE-0011-17). All analyses were conducted on anonymized data.

## 3. Results

### 3.1. Demographic Variables

The study included 100 participants with ABI, of whom 61 were diagnosed with dysphagia upon admission to rehabilitation and 39 presented without dysphagia. The 61 dysphagic patients included 46 (75.4%) participants dependent on enteral feeding alone (either nasogastric tube or percutaneous endoscopic gastrostomy) and 15 (24.6%) participants who had oral intake with limitations of different consistencies. The severity of dysphagia at admission, as graded according to FOIS, identified 40 (65.6%) participants with severe dysphagia, 4 (6.6%) participants with moderate dysphagia, and 17 (27.8%) participants with mild dysphagia. The average duration of the SLP treatment was 36 days among participants who recovered, compared to 136 days among those who did not (*p* < 0.001). Demographic characteristics for the two groups are presented in Table 1. No statistically significant differences were found between the groups in any of the analyzed demographic variables (age, gender and ethnicity).

### 3.2. Comparison of Etiology and Impairment Profile Between Dysphagic and Non-Dysphagic Participants

The etiology and impairment profile of the participants are presented in Table 1. TBI was the most common etiology among participants. Tumor of the CNS showed a tendency for association with dysphagia, although this did not reach statistical significance (*p* = 0.07), while participants with stroke had a significantly lower rate of dysphagia at admission to rehabilitation (*p* = 0.005) in comparison to other etiologies.

Overall, 67 (67%) of participants required mechanical ventilation during acute hospitalization, prior to rehabilitation admission (data not shown in tables). Among the dysphagia group, 47 (77%) participants were ventilated (OR = 3.5, *p* = 0.008) for a median duration of 10 days, compared to 20 (51.3%) participants in the non-dysphagia group, who were ventilated for a median of 5 days (*p* = 0.005). Participants with dysphagia had a significantly longer acute hospitalization, with a median of 28.5 days and IQR of [16.7, 25.6], compared to 16 days [13, 34] in the non-dysphagic group (*p* = 0.002). Similarly, the length of unconsciousness was greater in the dysphagia group, with a median of 10 days [1, 15] versus 1 day [0, 7] in the non-dysphagic group (*p* = 0.003). The incidence of pneumonia during acute hospitalization was not significantly higher in the dysphagia group (13.11% [n = 8]) compared to the non-dysphagia group (7.69% [n = 3]) (*p* = 0.52).

### 3.3. Associations Between Dysphagia at Admission and Demographic, Etiological and Impairment Profile

The following factors were found to be significantly associated with dysphagia at admission to rehabilitation: severity of TBI according to GCS (*p <* 0.001) with Cramer’s V = 0.6, which is considered a strong relation, duration of mechanical ventilation, (*p* = 0.005), duration of unconscious state (*p* = 0.003), and state of reduced consciousness at admission to rehabilitation (patients with a reduced conscious state had a 71.7 higher risk of dysphagia compared with their counterparts. (*p* = 0.001). Prior CNS surgery or injury to the base of the skull were not associated with an increased risk of dysphagia. The OR of dysphagia for each categorical variable is presented in Table 1.

### 3.4. Logistic Regression for Dysphagia at Admission to Rehabilitation

A multivariable logistic regression model was constructed to assess the associations of independent variables with dysphagia at admission, as described in the Methods section.

To predict dysphagia at admission to rehabilitation in our cohort of 100 patients, we employed Firth Logistic Regression using only independent variables that were found to be statistically significant at the level of *α* = 0.1.

The model was found to be statistically significant and demonstrates exceptionally high predictive accuracy. The results of the analysis, presented in Table 2, show that patients with reduced consciousness were 73 times more likely to present with dysphagia compared to patients with full consciousness (*p* < 0.001). Cranial nerve impairment increases the odds of dysphagia by 26-fold (*p* < 0.001), patients with voice disorders had a nearly 8-fold increase in odds for dysphagia (*p* < 0.001) and those with tumors have a 7.4-fold increase in odds for dysphagia (*p* = 0.017).

The Likelihood Ratio Chi square test confirmed the model’s superior fit compared to the null model (*p* < 0.001). While the wide 95% CIs for some odds ratios suggest a degree of precision instability due to the sample size, the overall strength and direction of the associations remain clinically significant. 

Nagelkerke R^2^ value of 0.706 indicates that the predictor may explain 70.6% of the likelihood of dysphagia at admission, which is considered very high given the small sample. The AUC was 0.928 (95% CI [0.88, 0.975]), indicating a high discrimination, which suggests that the model can correctly distinguish between patients with and those without dysphagia in nearly 93% of cases.

### 3.5. Recovery from Dysphagia at Discharge

At admission, 61 participants (61%) were diagnosed with dysphagia. Of these, 48 participants (78.68%) recovered from dysphagia by discharge. We examined variables associated with recovery from dysphagia.

Age, gender, and ethnicity were not found to be significantly different between participants who recovered from dysphagia and those who did not (Table 3).

Likewise, mechanical ventilation and/or duration of mechanical ventilation, length of hospitalization, or existence of pneumonia during acute hospitalization did not differ between the two groups. Existence of tracheostomy at admission to rehabilitation or a specific etiology of ABI were also not found to be associated with recovery.

The etiology and impairment profile of participants who recovered and who did not recover from dysphagia are presented in Table 3.

Variables found to be associated with recovery from dysphagia at discharge to rehabilitation included reduced conscious state (OR = 0.05, *p* < 0.001), enteral feeding at admission (OR = 0.08, *p* = 0.03), cranial nerve injury to hypoglossal (XII) nerve (OR = 0.065, *p* = 0.01), and severity of dysphagia. Of the 17 participants who had mild dysphagia, all recovered (100% recovery); of the 4 participants with moderate dysphagia, 3 recovered (75% recovery); and of the 40 participants with severe dysphagia, only 28 recovered (70% recovery). Accordingly, the likelihood of dysphagia recovery decreased with greater severity at admission (Cramer’s V = 0.325, *p* = 0.02; Table 3).

The variable ‘severity of dysphagia’ demonstrated a robust association with the recovery from dysphagia. However, the original distribution of this variable across three levels revealed a significant data sparsity issue, with only four participants classified as having moderate dysphagia. To rigorously evaluate the relationship between severity of dysphagia and recovery, we explored different category merging strategies. Initially, collapsing the moderate and severe categories resulted in a zero-frequency cell (zero patients with low severity who failed to recover), leading to an unstable model with an excessively wide CI for the OR. Conversely, merging the moderate and mild severity categories yielded balanced group sizes, eliminated zero-frequency cells, and produced a more precise and narrower CI for the OR between severity of dysphagia and recovery from dysphagia. Consequently, severity of dysphagia was dichotomized for the final analysis, comparing ‘severe’ disease with a combined ‘mild-to-moderate’ reference group. Given the strong association between severity of dysphagia and the primary outcome of recovery from dysphagia, the inter-relationships between severity of dysphagia and other clinical variables were further examined, as detailed in Table 4. The reported OR values represent the risk of presenting with severe disease for each evaluated variable.

Variables found to be associated with severity of dysphagia at admission to rehabilitation included bilateral CNS injury; mechanical ventilation and duration of mechanical ventilation; cranial nerve impairment to trigeminal (V), fascial nerve (VII), and glossopharyngeal and vagal nerves (IX, X), and hypoglossal nerve (XII); and reduced conscious state and length of reduced conscious state. Notably, CNS tumor was negatively associated with reduced severity of dysphagia.

Lower FOIS scores at admission significantly predicted a reduced probability of dysphagia recovery (Table 3).

### 3.6. Logistic Regression for Recovery from Dysphagia

A multivariable logistic regression model was constructed to assess the associations of independent variables with recovery from dysphagia in a cohort of 61 patients using Firth logistic regression.

The chosen model with the highest likelihood of dysphagia includes one variable and was found to be statistically significant, demonstrating a high predictive accuracy. The results of the analysis, presented in Table 5, show that patients diagnosed with dysphagia are almost 15 times more likely to recover if they are with intact consciousness state compared to patients with reduced consciousness (*p* < 0.001). However, the 95% CI length suggests some uncertainty while interpreting the clinical relevance of the findings.

Nagelkerke R^2^ value of 0.337 indicates that the predictor explains 33.7% of likelihood of recovery from dysphagia, which is considered reasonable given the small sample. The AUC was 0.784 (95% CI 0.66, 0.91), indicating good discrimination, which suggests that the model may correctly distinguish between patients with and those without dysphagia in over 78% of cases.

## 4. Discussion

The current study identified four clinical factors associated with the presence of dysphagia at admission to pediatric rehabilitation following ABI. Typically, in such small samples (n = 100), the Firth regression model includes fewer predictors. The fact that the final model includes four significant variables associated with the outcome suggests that these variables are strongly connected to dysphagia. Reduced consciousness at admission to rehabilitation, CNS tumor etiology, cranial nerve impairment and voice disorder, emerged as significant predictors. Reduced consciousness has previously been identified as a predictor of dysphagia in children with TBI [17]. Other variables previously found to be associated with dysphagia include impairment of cranial nerves (specifically CNS V, IX+X, and XII), which are all associated with innervation of the swallowing mechanism, and voice and speech impairments, both of which involve a system that shares muscular and neural structures with the swallowing mechanism [24].

Consistent with the prior literature, in both adults [14,16] and children [14], dysphagia in our cohort was also associated with markers of more severe illness, including longer duration of hospitalization and prolonged mechanical ventilation.

Regarding etiology, in the current study, CNS tumor at admission to rehabilitation was found to predict dysphagia, while stroke was found to be associated with reduced probability for dysphagia. In children, CNS tumors are mostly located in the posterior fossa and brain stem [20], which contain the anatomic regions of the cranial nerves responsible for the swallowing process. These regions are thus likely to be injured by tumor-related mass effect or surgical intervention leading to dysphagia. Addressing stroke etiology, our findings contrast with prior pediatric reports describing stroke as a common cause of dysphagia [25]. A possible explanation relates to lesion laterality. Right hemispheric stroke has been previously linked to dysphagia [26,27,28]. In the current study, however, of the eight children suffering stroke, none showed involvement of the right hemisphere. The reduced risk for dysphagia in these children may perhaps be explained by the involvement of the left hemisphere. Additionally, similar to findings by Liu et al. [15], we found bilateral CNS involvement to be associated with increased risk of dysphagia. Stroke involved one hemisphere in participants of the current study, while other etiologies leading to ABI like TBI, infection, or AnBI involved both hemispheres.

Interestingly, in addition to these variables, we also found an association of dysphagia with cognitive and behavioral impairments. Such impairments have not been previously reported to be associated with dysphagia in children with ABI. It is possible that in more severe injuries, a wide variety of functions are impaired, including that leading to dysphagia. This assumption may be supported by the association of dysphagia in our study, with other variables like bilateral CNS involvement, severity of TBI, and longer mechanical ventilation or hospitalization in the general hospital, which could all indicate increased severity of the primary injury. On the other hand, cognitive factors such as attention, praxis, and executive functions (planning, organization, and control) may also all contribute to the function of the swallowing mechanism. In adults, Dehaghani et al. [29] studied the association of cognition and attention impairments as mediators between brain lesions and dysphagia via three causal models. The authors proposed that dysphagia should not be defined solely on the basis of oral and pharyngeal movement disorders but may be a collaboration of at least two factors of orofacial motor disorders and cognitive dysfunction. Thus, according to the authors, cognitive functioning should be assessed in the diagnosis and treatment of dysphagia. Our results in pediatric ABI dysphagia are in congruence with the model proposed by Dehaghani and colleagues. Notably, however, although we found dysphagia at admission to be associated with cognitive and behavioral disturbances, these variables were not associated with lack of recovery. Further investigational studies should be carried out to explore the association of these factors with dysphagia at admission and with recovery from dysphagia.

Beyond predicting the occurrence of dysphagia, we examined factors associated with dysphagia severity. TBI etiology was positively associated with greater severity, while CNS tumor was negatively associated. Our findings align with studies by Morgan and colleagues [16,17], who demonstrated that the severity of TBI correlates with the severity of dysphagia. However, previous pediatric tumor studies in patients with dysphagia reported greater swallowing impairment following posterior fossa tumor resection [30,31]. This discrepancy between these findings may reflect differences in timing of rehabilitation referral. In the healthcare system used for the current study, children with CNS tumors are more likely to be referred to rehabilitation only after completion of radiation therapy and medical stabilization. In other health care settings, however, referral may occur earlier in the disease trajectory. Thus, the relatively lower dysphagia severity observed in our patients who had tumors may represent partial spontaneous recovery before admission to rehabilitation. In accordance with a previous study by Yogo and colleagues, we also demonstrated that mechanical ventilation and its duration are associated with the severity of dysphagia [32]. Bilateral CNS involvement was also associated with the severity of dysphagia, similar to findings by Liu and colleagues [15], most probably as an expression of the severity of the prior CNS injury.

In contrast to the limited data available in the literature regarding recovery from dysphagia in children with ABI, the majority of the participants in our study (78%) demonstrated recovery with specified SLP rehabilitative treatment. Non-recovery in our cohort was associated with reduced consciousness, cranial nerve XII impairment, and enteral feeding at admission. Logistic regression analysis indicated that conscious state explained 33.7% of the variance in recovery. Etiology of AnBI demonstrated a trend toward lower recovery rates, possibly reflecting small subgroup size. Notably, a previous study comparing long-term outcomes of pediatric TBI and AnBI [33] reported that children with AnBI had lower rate of consciousness regaining during rehabilitation, thus exhibiting lower rates of recovery from dysphagia, in comparison to children post TBI.

Additionally, greater dysphagia severity and enteral feeding at admission were also associated with lower likelihood of recovery. These findings are consistent with a study by Hansen et al. [34], who demonstrated that injury severity measured by GCS, Functional Independence Measure, and FOIS at admission were negatively associated with return to unrestricted oral intake in TBI patients. Likewise, D’Netto et al. reported that dysphagia severity in an adult post stroke population was able to predict non-recovery [35].

In summary, our findings suggest that dysphagia following pediatric ABI is multifactorial and closely linked to markers of neurological injury severity. Prolonged ventilation, cranial nerve impairment, bilateral CNS involvement, and reduced conscious state all appear to represent a more extensive neural disruption affecting swallowing networks. At the same time, cognitive factors may contribute to the clinical expression of dysphagia, supporting a multidimensional conceptualization that extends beyond isolated injury to anatomical brain regions subserving swallowing mechanism.

The current study has several limitations that should be acknowledged. First, the sample size was relatively small, which may limit the statistical power and the generalizability of the findings. In addition, the retrospective design introduces variability in data completeness and accuracy. More specifically, the instrumental swallowing assessments and severity scales other than FOIS were not consistently documented in the electronic medical files of the participants. Likewise, cognitive impairment was documented only according to the Dynamic-Occupational Therapy Cognitive Assessment for Children (D-OTCA-Ch) [23], while the emotional difficulties relied on clinical evaluation and not on a standardized assessment tool. The cohort included children with mixed etiologies, potentially obscuring etiology-specific patterns of dysphagia. Although the predominance of male participants reflects the known higher incidence of males secondary to traumatic injury [36], this sex imbalance may still restrict the wider applicability of the results. Furthermore, because patients were referred to rehabilitation from multiple hospitals, some clinical variables—particularly the documentation of complications during prior hospitalization, their severity, and their temporal relationship to dysphagia—were not reported in a standardized manner, introducing inter-institutional reporting biases. Another important limitation of the study is that due to lack of a control group that did not receive treatment, it is difficult to disentangle the effects of the therapeutic interventions from spontaneous recovery, especially in the type of population studied, in which neuroplastic changes are expected over time.

Future research should address these limitations by employing larger, prospectively designed studies with standardized data collection across centers, control groups, and long-term follow-up, to better characterize recovery trajectories and treatment effects. Despite these limitations, the present study provides valuable preliminary evidence in an under-researched population and contributes clinically relevant insights to the understanding of predictors of pediatric dysphagia following ABI.

## 5. Conclusions

In conclusion, dysphagia is a common impairment in children following ABI admitted to rehabilitation. Recovery rates with assigned rehabilitative swallowing treatment by SLP are high. Defining prognostic factors at admission and discharge may guide clinicians in allocating the proper treatment and duration of care, as well as assist them in counseling families about the course of recovery.

## Figures and Tables

**Table 1:** Demographic variables, etiology and impairment profile at admission by group (dysphagia [n = 61] and non-dysphagia [n = 39]) and in the total group of participants (n = 100).

Categorical Variables		Total (n = 100)	Dysphagia (n = 61)		Non-Dysphagia (n = 39)		*p*	Effect Size: OR [95% CI]
		%	%	n	%	n		
Demographic	Gender (male)	70	65.6	40	30	76.9	0.23	1.75 [0.71, 4.36]
	Ethnicity (Jewish)	28	31	18	10	25.6	0.56	0.77 [0.31, 1.9]
Etiology	TBI	61	62.3	38	59	23	0.74	1.15 [0.50, 2.61]
	CNS tumor	16	21.7	13	7.7	3	0.07	3.25 [0.86, 12.2]
	Stroke	8	1.6	1	17.9	7	0.005	0.07 [0.009,0.65]
	AnBI	7	9.8	6	2.6	1	0.24	4.14 [0.48, 35.8]
	Infections	5	3.3	2	10.3	4	0.15	0.3 [0.05, 1.7]
	ADEM	3	1.6	1	5.1	2	0.56	0.31 [0.03, 3.52]
Impairment at admission to rehabilitation	Reduced conscious state	29	47.5	29	0	0	<0.001	71.7 [9.4, 9228]
	Motor impairment	99	100	61	97.4	38	0.39	4.8 [0.25, 706.9]
	Cognitive impairment *	93	98.4	60	84.6	33	0.013	10.9 [1.3, 64.5]
	Behavioral impairment	56	68.5	37	48.7	19	0.054	2.3 [0.98, 5.36]
	Bilateral CNS injury	65	75.4	46	51.4	19	0.016	2.9 [1.2, 6.9]
	Speech disorders	61	80.3	49	30.8	12	<0.001	9.2 [3.6, 23.3]
	Voice disorders	48	65.6	40	20.5	8	<0.001	7.4 [2.9, 18.9]
	Tracheostomy	11	14.75	9	5.1	2	0.19	3.2 [0.65, 15.7]
Variable occurring prior to rehabilitation	Mechanical ventilation	68	78.7	48	51.3	20	0.008	3.5 [1.5, 8.4]
	Pneumonia	11	13.11	8	0.77	3	0.518	1.81 [0.45, 7.29]
Variable detected at admission to rehabilitation	Cranial nerve impairment (total)	85	98.4	60	64.1	25	<0.001	33.6 [4.2, 269.4]
	V	47	63.3	38	33.3	9	0.01	3.5 [1.3, 8.9]
	VII	72	85	51	77.8	21	0.54	1.62 [0.51, 5.1]
	IX + X	50	75	45	18.5	5	<0.001	31.2 [4.25, 41]
	XII	46	71.7	43	11.1	3	<0.001	20.2 [5.4, 76.1]
Quantitative variables, median [IQR]								Effect size: r
Age in years		12 [7, 15]	12 [6, 14.5]		13 [9.5, 16]		0.16	0.14
Days from injury to rehabilitation		26 [15, 47]	28 [16, 52]		16 [13, 34]		0.002	0.316
Duration of reduced consciousness in days		3 [0, 12]	10 [1, 15]		1 [0, 7]		0.003	0.297
Duration of mechanical ventilation in days		8 [4, 15]	10 [5, 15]		5 [3, 7]		0.005	0.298

OR = odds ratio; ADEM = acute disseminated encephalomyelitis; AnBI = anoxic brain injury; CI = confidence interval; CNS = central nervous system; IQR = interquartile range; TBI = traumatic brain injury. * Cognitive disturbances were evaluated using the Dynamic-Occupational Therapy Cognitive Assessment for Children (D-LOTCA-Ch) [23].

**Table 2:** Results of a Firth logistic regression model for effect on participant’s probability for dysphagia at admission (n = 100).

Variable	Odds Ratio	95% CI	*p*
Intercept	0.013	(0.005, 0.12)	<0.001
Reduced conscious state at admission	73.63	(8.54, 974.1)	<0.001
Cranial nerve impairment	26.34	(3.42, 534.33)	<0.001
Voice disorders	7.9	(2.43, 29.64)	<0.001
Tumors	7.43	(1.4, 75.49)	0.017
Likelihood Ratio Chi square (4) = −66.04, *p* < 0.001 Nagelkerke R^2^ = 0.706, AUC 0.928, 95% CI [0.88, 0.975]			

AUC = area under the curve; CI = confidence interval.

**Table 3:** Demographic variables, etiology, and impairment profile at admission to rehabilitation and prior to admission to rehabilitation of participants with dysphagia (n = 61), those who recovered from dysphagia (n = 48), and those who did not recover from dysphagia (n = 13).

Categorical Variables		Total (n = 61)		Recovery (n = 48)		Non-Recovery (n = 13)		*p*	Effect Size: OR [95% CI]
		%	n	%	n	%	n		
Demographic	Gender (male)	65.6	40	70.8	34	46.2	6	0.23	0.35 [0.1, 1.24]
	Ethnicity (Jewish)	31	18	28.9	13	38.5	5	0.51	1.54 [0.42, 5.59]
Etiology	TBI	62.3	38	62.5	60	61.5	8	0.99	1.04 [0.29, 3.67]
	CNS tumor	21.3	13	22.9	11	15.4	2	0.72	1.63 [0.31, 8.51]
	Stroke	1.6	1	2.1	1	0	0	0.99	0.85 [0.04, 127.3]
	AnBI	9.8	6	6.3	3	23.1	3	0.1	0.22 [0.04, 1.27]
	Infections	3.3	2	4.2	2	0	0	0.99	1.45 [0.11, 204]
	ADEM	1.6	1	2.1	1	0	0	0.99	0.85 [0.04, 127.3]
Impairment at admission to rehabilitation	Reduced conscious state	47.5	29	35.4	17	92.3	12	<0.001	0.05 [0.005, 0.38]
	Cognitive impairment	98.4	60	97.9	47	100	13	0.99	1.17 [0.09, 23.3]
	Behavioral impairment	68.5	37	66	31	85.7	6	0.41	0.32 [0.04, 2.92]
	Bilateral CNS injury	75.4	46	75	36	76.9	10	0.99	0.9 [0.21, 3.82]
	Speech disorders	80.3	49	79.2	38	84.6	11	0.99	0.69 [0.13, 3.63]
	Voice disorders	65.6	40	66.7	32	61.5	8	0.75	1.25 [0.35, 4.44]
	Enteral Feeding (at admission to rehabilitation)	75.4	46	68.8	33	100	13	0.03	0.08 [0.006, 0.68]
	Tracheostomy	14.8	9	12.5	6	23.1	3	0.78	0.48 [0.11, 2.23]
	Cranial nerves impairment	98.4	60	97.9	47	100	13	0.99	1.17 [0.09, 23.3]
Variable occurring prior to rehabilitation	Mechanical ventilation	78.7	48	79.2	38	76.9	10	0.99	1.14 [0.26, 4.9]
	Pneumonia	13.1	8	12.5	6	15.4	2	0.99	0.78 [0.14, 4.44]
Variables detected at admission to rehabilitation	V	63.3	38	57.4	27	84.6	11	0.1	0.24 [0.05, 1.23]
	VII	85	51	85.1	40	84.6	11	0.99	1.04 [0.19, 5.73]
	IX+X	75	45	72.3	34	84.6	11	0.48	0.48 [0.09, 2.44]
	XII	71.7	43	63.8	30	100	13	0.01	0.065 [0.005, 0.54]
Severity of dysphagia	Mild (FOIS 6)	27.9	17	35.4	17	0	0	0.02	0.325 ^a^
	Moderate (FOIS 4–5)	6.6	4	6.3	3	7.7	1		
	Severe (FOIS 1–3)	65.6	40	58.3	28	92.3	12		
Quantitative variables		Median [IQR]						*p*	Effect size: r
Age in years		12 [6, 14.5]		12 [6, 15]		12 [10, 14]		0.71	0.04
Days from injury to rehabilitation		28 [16, 52]		28 [16, 52]		31 [24, 66]		0.53	0.08
Duration of reduced conscious in days		10 [1, 15]		5 [1, 14]		30 [2, 60]		0.16	0.20
Duration of mechanical ventilation in days		10 [5, 15]		10 [5, 15]		10 [5, 31]		0.46	0.11

OR = odds ratio; ADEM = acute disseminated encephalomyelitis; AnBI = anoxic brain injury; CNS = central nervous system; IQR = interquartile range; TBI = traumatic brain injury. FOIS = Functional Oral Intake Scale; ^a^ Cramer’s V value.

**Table 4:** Demographic, etiology, and impairment profile at admission to rehabilitation and prior to admission to rehabilitation by severity of dysphagia (severe (n = 40), mild or moderate (n = 21) as reference) among patients diagnosed with dysphagia at admission to rehabilitation (n = 61).

∫		Total (n = 61)		Severe (n = 40)		Mild or Moderate (n = 21)		*p*	Effect Size: Odds Ratio [95% CI]
		%	n	%	n	%	n		
Demographic	Gender (male)	65.6	40	67.5	27	61.9	13	0.66	1.28 [0.42, 3.84]
	Ethnicity (Jewish)	31	18	32.4	12	28.6	6	0.76	1.2 [0.37, 3.87]
Etiology	TBI	62.3	38	70	28	47.6	10	0.1	2.56 [0.86, 7.63]
	CNS tumor	21.3	13	12.5	5	38.1	8	0.045	0.23 [0.06, 0.84]
	Stroke	1.6	1	2.5	1	0	0	0.99	1.63 [0.08, 242.9]
	AnBI	9.8	6	12.5	5	4.8	1	0.65	2.86 [0.31, 26.31]
	Infections	3.3	2	2.5	1	4.8	1	0.99	0.53 [0.03, 8.62]
	ADEM	1.6	1	0	0	4.8	1	0.34	0.17 [0.001, 3.3]
Impairment at admission to rehabilitation	Reduced conscious state (at admission)	47.5	29	72.5	29	0	0	<0.001	111.1 [13.2, 14.443]
	Cognitive impairment	98.4	60	100	40	95.2	20	0.34	5.93 [0.3, 880.9]
	Behavioral impairment	68.5	37	75.8	25	57.1	12	0.23	2.34 [0.72, 7.57]
	Bilateral CNS injury	75.4	46	85	34	57.1	12	0.02	4.25 [1.25, 14.5]
	Speech disorders	80.3	49	85	34	71.4	18	0.31	2.27 [0.63, 8.19]
	Voice disorders	65.6	40	62.5	25	71.4	15	0.49	0.67 [0.22, 2.09]
	Tracheostomy	14.8	9	17.5	7	9.5	2	0.48	2.01 [0.38, 10.75]
	Cranial nerves impairment	98.4	60	100	40	95.2	20	0.34	5.93 [0.3, 880.9]
Variable occurring prior to rehabilitation	Mechanical ventilation	78.7	48	87.5	35	61.9	13	0.045	4.3 [1.2, 15.6]
	Pneumonia	13.1	8	15	6	9.5	2	0.70	1.68 [0.31, 9.17]
Variables detected at admission to rehabilitation	V	63.3	38	77.5	31	35	7	0.002	6.41 [1.96, 20.83]
	VII	85	51	92.5	37	70	14	0.049	5.29 [1.16, 23.8]
	IX+X	75	45	85	34	55	11	0.02	4.63 [1.34, 15.87]
	XII	71.7	43	85	34	45	9	0.001	6.94 [2.01, 23.8]
Quantitative variables, median [IQR]								*p*	Effect size: r
Age, in years		12 [6, 14.5]		13 [8, 15]		8 [5, 13]		0.16	0.18
Days from injury to rehabilitation		28 [16, 52]		30 [19, 66]		26 [15, 48]		0.19	0.17
Duration of reduced conscious in days		10 [1, 15]		14 [5, 30]		1 [0, 4]		0.002	0.44
Duration of mechanical ventilation in days		10 [5, 15]		11 [7, 17]		4 [0, 10]		0.03	0.33

ADEM = acute disseminated encephalomyelitis; AnBI = anoxic brain injury; CI = confidence interval; CNS = central nervous system; IQR = interquartile range; TBI = traumatic brain injury.

**Table 5:** Results of a Firth logistic regression model for effect on participant’s probability for recovery from dysphagia at discharge (n = 61).

Variable	Odds Ratio	95% CI	*p*
Intercept	1.4	(0.68, 2.96)	0.36
Consciousness at admission	14.98	(3.19, 146.1)	<0.001
Likelihood Ratio Chi square (1) = −13.78, *p* < 0.001 Nagelkerke R^2^ = 0.337, AUC 0.784, 95% CI [0.66, 0.91]			

AUC = area under the curve; CI = confidence interval.

## Data Availability

All data supporting the findings of this study are available upon request from the corresponding authors.

## Abbreviations

The following abbreviations are used in this manuscript:

ABI	Acquired Brain Injury
ADEM	Acute disseminated encephalomyelitis
AnBI	Anoxic brain injury
CNS	Central nervous system
FOIS	Functional Oral Intake Scale
GCS	Glasgow Coma Scale
TBI	Traumatic brain injury

## References

[1] Halfpenny R., Stewart A., Kelly P., Conway E., Smith C. (2021). Dysphagia Rehabilitation Following Acquired Brain Injury, Including Cerebral Palsy, across the Lifespan: A Scoping Review Protocol. Syst. Rev..

[2] Turner-Stokes L., Pick A., Nair A., Disler P.B., Wade D.T. (2015). Multi-Disciplinary Rehabilitation for Acquired Brain Injury in Adults of Working Age. Cochrane Database Syst. Rev..

[3] Anderson V., Brown S., Newitt H., Hoile H. (2009). Educational, Vocational, Psychosocial, and Quality-of-Life Outcomes for Adult Survivors of Childhood Traumatic Brain Injury. J. Head Trauma Rehabil..

[4] Kumar S., Doughty C., Doros G., Selim M., Lahoti S., Gokhale S., Schlaug G. (2014). Recovery of Swallowing after Dysphagic Stroke: An Analysis of Prognostic Factors. J. Stroke Cerebrovasc. Dis..

[5] Attrill S., White S., Murray J., Hammond S., Doeltgen S. (2018). Impact of Oropharyngeal Dysphagia on Healthcare Cost and Length of Stay in Hospital: A Systematic Review. BMC Health Serv. Res..

[6] Alhashemi H.H. (2010). Dysphagia in Severe Traumatic Brain Injury. Neurosciences.

[7] Geeganage C., Beavan J., Ellender S., Bath P.M. (2012). Interventions for Dysphagia and Nutritional Support in Acute and Subacute Stroke. Cochrane Database Syst. Rev..

[8] Leder S.B., Suiter D.M. (2009). An Epidemiologic Study on Aging and Dysphagia in the Acute Care Hospitalized Population: 2000–2007. Gerontology.

[9] Becker R., Nieczaj R., Egge K., Moll A., Meinhardt M., Schulz R.-J. (2011). Functional Dysphagia Therapy and PEG Treatment in a Clinical Geriatric Setting. Dysphagia.

[10] McMicken B.L., Muzzy C.L. (2009). Prognostic Indicators of Functional Outcomes in First Time Documented Acute Stroke Patients Following Standard Dysphagia Treatment. Disabil. Rehabil..

[11] Schindler A., Vincon E., Grosso E., Miletto A.M., Di Rosa R., Schindler O. (2008). Rehabilitative Management of Oropharyngeal Dysphagia in Acute Care Settings: Data from a Large Italian Teaching Hospital. Dysphagia.

[12] Ward E.C., Green K., Morton A.-L. (2007). Patterns and Predictors of Swallowing Resolution Following Adult Traumatic Brain Injury. J. Head Trauma Rehabil..

[13] Mangilli L.D., Sassi F.C., De Medeiros G.C., De Andrade C.R.F. (2012). Rehabilitative Management of Swallowing and Oral-Motor Movements in Patients with Tetanus of a Public Service in Brazil. Acta Trop..

[14] Starks B., Harbert C. (2011). Aspiration Prevention Protocol: Decreasing Postoperative Pneumonia in Heart Surgery Patients. Crit. Care Nurse.

[15] Liu C.H., Huo M., Qin H.H., Zhao B.L. (2022). Critical Prognostic Factors for Poststroke Dysphagia: A Meta-Analysis. Eur. Rev. Med. Pharmacol. Sci..

[16] Morgan A.T., Mageandran S.-D., Mei C. (2010). Incidence and Clinical Presentation of Dysarthria and Dysphagia in the Acute Setting Following Paediatric Traumatic Brain Injury. Child Care Health Dev..

[17] Morgan A., Ward E., Murdoch B., Kennedy B., Murison R. (2003). Incidence, Characteristics, and Predictive Factors for Dysphagia after Pediatric Traumatic Brain Injury. J. Head Trauma Rehabil..

[18] Morgan A.B., Ward E., Murdoch B., Bilbie K.B. (2002). Acute Characteristics of Pediatric Dysphagia Subsequent to Traumatic Brain Injury: Videofluoroscopic Assessment. J. Head Trauma Rehabil..

[19] Sherman V., Martino R., Bhathal I., DeVeber G., Dlamini N., MacGregor D., Pulcine E., Beal D.S., Thorpe K.E., Moharir M. (2021). Swallowing, Oral Motor, Motor Speech, and Language Impairments Following Acute Pediatric Ischemic Stroke. Stroke.

[20] Millard N.E., De Braganca K.C. (2016). Medulloblastoma. J. Child Neurol..

[21] Anestis D.M., Marinos K., Tsitsopoulos P.P. (2023). Comparison of the Prognostic Validity of Three Simplified Consciousness Assessment Scales with the Glasgow Coma Scale. Eur. J. Trauma Emerg. Surg..

[22] Crary M.A., Mann G.D.C., Groher M.E. (2005). Initial Psychometric Assessment of a Functional Oral Intake Scale for Dysphagia in Stroke Patients. Arch. Phys. Med. Rehabil..

[23] Katz N., Golstand S., Bar-Ilan R.T., Parush S. (2007). The Dynamic Occupational Therapy Cognitive Assessment for Children (DOTCA–Ch): A New Instrument for Assessing Learning Potential. Am. J. Occup. Ther..

[24] Groher M.E., Crary M.A. (2020). Dysphagia-E-Book: Clinical Management in Adults and Children.

[25] Dodrill P., Gosa M.M. (2015). Pediatric Dysphagia: Physiology, Assessment, and Management. Ann. Nutr. Metab..

[26] Falsetti P., Acciai C., Palilla R., Bosi M., Carpinteri F., Zingarelli A., Pedace C., Lenzi L. (2009). Oropharyngeal Dysphagia after Stroke: Incidence, Diagnosis, and Clinical Predictors in Patients Admitted to a Neurorehabilitation Unit. J. Stroke Cerebrovasc. Dis..

[27] Robbins J., Levine R.L., Maser A., Rosenbek J.C., Kempster G.B. (1993). Swallowing after Unilateral Stroke of the Cerebral Cortex. Arch. Phys. Med. Rehabil..

[28] Suntrup S., Kemmling A., Warnecke T., Hamacher C., Oelenberg S., Niederstadt T., Heindel W., Wiendl H., Dziewas R. (2015). The Impact of Lesion Location on Dysphagia Incidence, Pattern and Complications in Acute Stroke. Part 1: Dysphagia Incidence, Severity and Aspiration. Eur. J. Neurol..

[29] Ebrahimian Dehaghani S., Yadegari F., Asgari A., Bagheri Z. (2019). The Mediator Effect of Cognition on the Relationship between Brain Lesion Location and Dysphagia in Patients with Stroke: Applying a Structural Equation Model. J. Oral Rehabil..

[30] Goethe E.A., Gadgil N., Stormes K., Wassef A., LoPresti M., Lam S. (2020). Predicting Dysphagia in Children Undergoing Surgery for Posterior Fossa Tumors. Child’s Nerv. Syst..

[31] Wright S.H., Blumenow W., Kumar R., Mallucci C., Felton A., McMahon S., Hennigan D., Avula S., Pizer B. (2023). Prevalence of Dysphagia Following Posterior Fossa Tumour Resection in Children: The Alder Hey Experience. Child’s Nerv. Syst..

[32] Yogo N., Abe T., Kano K., Muto Y., Kiyonaga S., Hirai K. (2024). Post-Extubation Dysphagia in Pediatric Trauma Patients: A Single-Center Case-Series Study. Sci. Rep..

[33] Shaklai S., Peretz Fish R., Simantov M., Groswasser Z. (2018). Prognostic Factors in Childhood-Acquired Brain Injury. Brain Inj..

[34] Hansen T.S., Engberg A.W., Larsen K. (2008). Functional Oral Intake and Time to Reach Unrestricted Dieting for Patients With Traumatic Brain Injury. Arch. Phys. Med. Rehabil..

[35] D’Netto P., Rumbach A., Dunn K., Finch E. (2023). Clinical Predictors of Dysphagia Recovery After Stroke: A Systematic Review. Dysphagia.

[36] Thurman D.J. (2014). The Epidemiology of Traumatic Brain Injury in Children and Youths: A Review of Research Since 1990. J. Child Neurol..

